# PSSNet—An Accurate Super-Secondary Structure for Protein Segmentation

**DOI:** 10.3390/ijms232314813

**Published:** 2022-11-26

**Authors:** Denis V. Petrovsky, Vladimir R. Rudnev, Kirill S. Nikolsky, Liudmila I. Kulikova, Kristina M. Malsagova, Arthur T. Kopylov, Anna L. Kaysheva

**Affiliations:** Biobanking Group, Branch of Institute of Biomedical Chemistry “Scientific and Education Center”, 109028 Moscow, Russia

**Keywords:** super-secondary structure, data bank, AlphaFold 2.0, graph neural network, machine learning, protein features

## Abstract

A super-secondary structure (SSS) is a spatially unique ensemble of secondary structural elements that determine the three-dimensional shape of a protein and its function, rendering SSSs attractive as folding cores. Understanding known types of SSSs is important for developing a deeper understanding of the mechanisms of protein folding. Here, we propose a universal PSSNet machine-learning method for SSS recognition and segmentation. For various types of SSS segmentation, this method uses key characteristics of SSS geometry, including the lengths of secondary structural elements and the distances between them, torsion angles, spatial positions of Cα atoms, and primary sequences. Using four types of SSSs (βαβ-unit, α-hairpin, β-hairpin, αα-corner), we showed that extensive SSS sets could be reliably selected from the Protein Data Bank and AlphaFold 2.0 database of protein structures.

## 1. Introduction

Protein folding mechanisms have fascinated scientists for a half of a century [1,2,3]. According to the “nucleation–condensation” model of protein folding, self-folding proteins, such as molecular chaperones [4], that do not participate in the protein machinery, become unstructured tangles immediately after translation. Folding nuclei (a time-limiting stage) are formed and condensed in coils, and the process is completed by spontaneous packing into a native three-dimensional structure [5,6,7]. In relation to this concept, attention has been focused toward simple motifs such as super-secondary structures (SSSs) that comprise several secondary structure elements with unique and compact folding of a polypeptide chain. Super-secondary structures serve as a bridge between the secondary and tertiary structure of a protein and probably are autonomously stable (i.e., stable outside the protein globule) [8,9].

The use of SSSs to solve biomedical problems is rather desirable, as the alpha-helical and beta-hairpin types of SSSs can serve as initial unique structures for the construction of protein epitope mimetics (PEMs) [10,11]. These PEMs mimic the structural and conformational properties of their target epitopes (SSS), as well as their biological activity (protein–protein and protein–nucleic acid interactions). It is possible to optimize biological activity to maintain antimicrobial activity, for example, by transferring an epitope from a recombinant to a synthetic scaffold [11].

In previous studies, we reported the possibility of studying SSSs in aberrant protein forms caused by post-translational modifications (PTMs). We observed that PTMs that have been detected in patients with various types of cancer are frequently localized in the SSS (alpha-structural motifs, beta-hairpins) [12]. So far, it is obvious that a comprehensive study of the known SSS types is essential for deeper insights of protein folding mechanisms and to solve some challenges in biomedical research [9].

Predicting the secondary and supersecondary structures of proteins by their 3D-structures (PDB, AlfaFold) is becoming a top priority in structural biology research. Numerous approaches for prediction are currently known but the most commonly used are based on (1) probabilistic models, such as kernel density estimation (KDE) [13,14] and naïve Bayes [15]; (2) linear classifiers, such as support vector machines (SVM) [16,17,18]; and (3) machine learning methods [9,19]. The performance of the first two approaches is limited by the huge amounts of data with relatively low classification and semantic segmentation accuracy (60–75%) [16,20].

Neural networks (NN) have recently been applied to the problem of structures classification and segmentation. Neural networks are typically designed to classify and/or predict one or two types of SSSs, though several NN-utilizing methods are now capable of predicting β-hairpin and βαβ-units (StackSSSPred) [21,22,23]. The following main groups of machine learning models are most widely used:

1. Models based on the sequence-to-sequence architecture, where the protein structure is considered as a sequence of amino acids with the main characteristics of their localization, i.e., contact map. Featured sequences are processed using a group of recurrent layers [24].
2. Models based on 3D-CloudSegmentation, in which each atom of a molecule is represented as a point in a 3D space. PointNet, PointNet++, and dynamic graph CNN (DGCNN) architectures [25] are used to segment and classify structures.
3. Models based on the representation of a protein molecule as a 3D volumetric object (protein voxelization) with subsequent processing by 3D-Convolution family networks [26].
4. Models based on the representation of a protein molecule as a graph with subsequent processing by graph neural networks (CGNs) [27].

Here, we present a new approach to classify different types of SSSs, specifically βαβ-unit, α-hairpin, β-hairpin, and αα-corner, and the approach was tested on standard format files extracted from the public Protein Data Bank (PDB) [9]. The neural network PSSNet (Protein Secondary Structure Segmentation) was realized on a new deep learning architecture that uses the integrative synergy of CGN, convolutional neural networks (CNN), and (bidirectional) recurrent neural network (RNN) predictions. The proposed architecture achieves an accuracy of 84% and endorses a wide range of valuable annotations for over 1.9 million SSSs available in the open-access knowledge base at https://psskb.org/ (accessed on 28 October 2022). In addition to the secondary structure prediction, PSSNet can also be applied for the prediction of free energy, solvent availability, contact maps, and searching for stable protein structures.

## 2. Results and Discussion

### 2.1. SSS Segmentation Using the PSSNet Model

This model was the basis for the filling of gaps in the open knowledge base of SSSs (available at https://psskb.org/, accessed on 28 October 2022). After training the model, we applied it to complete dataset maintained in the PDB and AlphaFold. The results were selectively assessed by expert researchers, entered into a database, and finalized as publicly available. The number of SSSs defined by the model are listed in Table 1.

The model was built according to the proposed architecture and combined high accuracy and performance. We assessed the performance of the model engine by comparing its quality with that of networks with distinct architectures, i.e., CurveNet [28] and DGCNN36 networks (Table 2). Such networks are among the top 10 utilized in “3D point cloud” classification and segmentation [29]. Training and evaluation of the results were carried out on the same datasets. Plots of the loss function and IOU versus iterations are provided in Supplementary Materials (model evaluation metrics section).

The predictive power of a machine learning model is mainly determined by its feature representation and feature extraction algorithms. The models considered in our comparative experiment operate only with the 3D coordinates of protein atoms, however these features were barely enough to provide acceptable recognition accuracy (Table 2). In contrast, our model operates with an extended set of specific structural features, encompassing torsion angles, the spatial positions of atoms in the amino acid sequence, and the primary protein sequence. The convolutional filters of our neural network blocks capture and generalize the local geometric features of the protein sequence well, and the subsequent blocks of Bi-GRUs capture the features of the global feature context.

### 2.2. Practical Evaluation of the Model: Key Issues

The proposed model was empirically tested to assess its accuracy and discover key objections that might arise from structure segmentation and classification. A random sample of 5000 SSS structures from PDB and AlphaFold was consolidated, and topology compliance with the studied motifs was examined.

**Figure 1:**
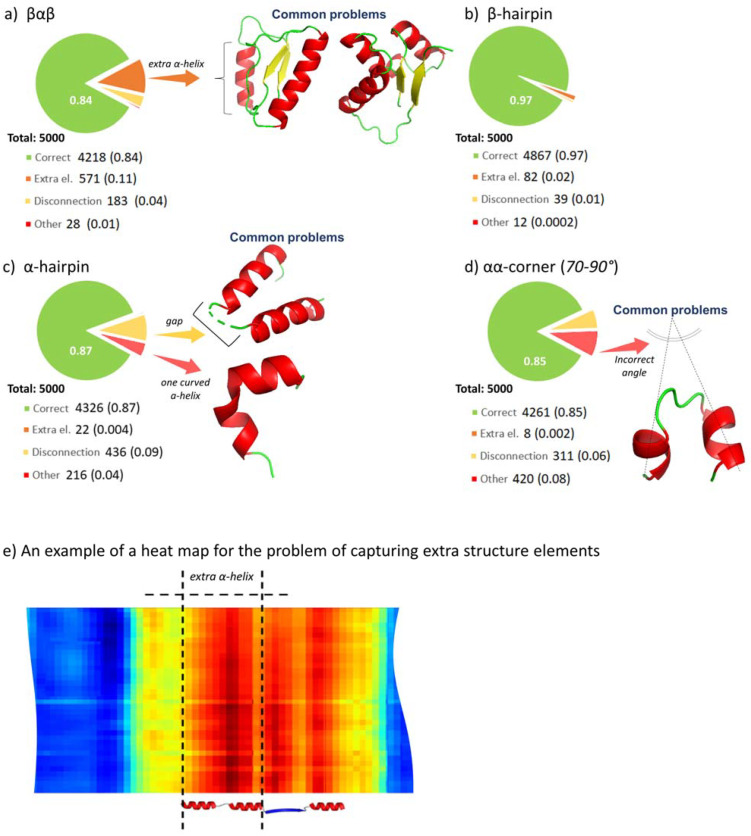
Evaluation of the model on empirical consolidated datasets (random sampling of 5000 elements for each type of SSS) of (**a**) βαβ-unit, (**b**) β-hairpin, (**c**) α-hairpin and (**d**) αα-corner, (**e**) heat map for the problem of extra α-helix.

The main issues that arose from the classification and segmentation of structural motifs were as follows (Figure 1):

(a) Capture of excess structure sections;
(b) Breakages in structure element links;
(c) Incorrect definition of corners (typical for the αα-corner motif (70°–90°)).

Despite the results of the model validation being quite satisfying (IoU = 0.92), the estimated accuracy ranged between 0.83–0.85. Thus, we established several problems specific to the topology of a specific type of SSS.

- Capturing extra sections is a prerequisite for SSS with α-helix elements, and it usually manifests if the distance between the last and first Cα atoms of the first and the second structures, respectively, is 9.7 Å. However, the network at the output from the GRU layer generates a feature map that captures both structures (Figure 1e). This issue was fixed by reducing the number of neighbors in the knn-graph, when generating features, or by producing a sufficiently larger sample of such structures and subsequently retraining the model. The identification and extraction of such elements from the PSSKB database are currently ongoing.
- Breakages in structures generally occurred in low-resolution (>4.0 Å) PDB files, but the proportion was insignificant and relatively narrow compared to the total size of the consolidated databank.
- The network identified curved helices with a large angle of inflection as two elements with incorrectly defined angles for αα-corner structures. Rigorous analysis revealed that the issue can be effectively resolved only if we performed retraining of the model on a meaningfully larger representative sample that covers all such elements; the retraining and sample collecting are currently in progress.

Despite the difference in accuracy between the actual and validation datasets, the model managed SSS segmentation and classification tasks well. The difference in accuracy suggests relatively high folding variability among the structures downloaded from PDB and AlphaFold. Hence, the training dataset must be sufficiently extended, especially in terms of negative examples, to improve the accuracy of segmentation and classification.

The composition of super-secondary structures is simple combinations of α-helices, β-hairpins, and short loops with a well-defined hydrophobic core involved in SSS stabilization. The loop–helix, β-hairpin, and Greek key motifs are prominent representatives of SSSs [30,31,32]. Characterization of such structures allows us to collect a catalog of autonomously stable protein motifs and archetypes [30,31]. These structures also serve as promising objects of study in protein physics (the study of folding), bioengineering (the development of peptide mimetics), and biomedicine (the study of conformational changes in aberrant forms of proteins compared with intact forms).

Medical proteomic research is mainly focused on the extensive study of the molecular basis of a disease associated with the arrival of aberrant forms of proteins that are regularly not found under normal (healthy) conditions. Aberrant proteins are frequently caused by genetic polymorphisms, alternative splicing, and PTMs [33,34]. Such structural changes associated with the disease can be localized in different types of SSSs. Numerous aberrant forms of proteins are fraught with dire structural changes, including isomers of beta-amyloid in Alzheimer’s disease [35], splice isoforms of osteopontins b and c in prostate cancer [36], amino acid substitutions in protein C7 in type II diabetes [37], and PTMs of proteins in oncological diseases [12].

Here, we present a new approach to frame the problem of SSS recognition and segmentation based on the geometric characteristics of structures and spatial relationships within a protein sequence. The main advantage of our method is a low requirement for computing sources. We used a standard personal desktop computer with a typical GeForce GTX 1650—4Gb video card for training and data processing in the PDB and AlphaFold2.0 databases. We also operated the PSSNet model with high recognition accuracy (mean IoU > 0.84; F1 > 0.08) and annotation capability of >1.9 million SSSs of βαβ-unit hairpin, β-hairpin, and αα-corner. This opens up wide margins for investigation on the PSSKB resource. The model does not require a large training set, since sets of 2000 specimens were used to train the model. Plots of GPU memory usage as a function of protein sequence length can be found in Supplementary Materials (GPU memory usage section).

A distinctive feature of our model is its ability to recognize and segment the SSSs within a protein sequence of arbitrary length, i.e., regardless of the sequence length. The model can operate directly with any file in PDB format, including those with low data quality, poor resolution, and sparse protein sequences. The only limitation of the model is the amount of graphics processing unit (GPU) memory. Likewise, most current models focus on recognition of only one certain type of structure and work only with a few prepared datasets, resulting in relatively low numbers of recognized structures within the range of thousands to tens of thousands.

The architecture of the proposed method is powered by a comprehensive combination of CGNs, CNNs, Bi-GRUs, self-attention, and multi-head attention mechanisms, which encourage network flexibility and easy adaptation to solve problems in structural biology and bioinformatics. Primary examinations have shown that a model with minimal modifications can predict the structural alphabet based on geometric characteristics for differentiable molecular modeling problems. A subsequent investigation will target this and other issues.

## 3. Materials and Methods

### 3.1. Data Preparation

Training, test, and validation datasets downloaded from the Protein Data Bank were represented by the following types of SSSs: a βαβ-motif (beta-alpha-beta motif), a β-hairpin, an α-hairpin, and an αα-corner (70°–90°).

The datasets were generated using STRIDE, which takes a PDB file as input and returns secondary structure assignments. Thereafter, data were manually curated by a team of experts to ensure compliance with the declared types of SSS. Eventually, the sets of positive and negative examples included almost 2000 and 4000 elements of SSS of each type. Training and test model datasets are available at https://doi.org/10.6084/m9.figshare.21529812.v1 (open access, accessed on 28 October 2022) [38].

The balance between positive and negative examples in packets supplied to the network input was regulated by the software implementation. Before entering the network, the coordinates of atoms *x*, *y*, and *z* were augmented (rotation around the *x*, *y*, and *z* axes at a random angle and the *y*-axis with random jitter for each point using Gaussian noise with zero-mean value and standard deviation of 0.08). Data augmentation was executed dynamically during the training time for 40% of input structures.

Before entering the network, elements of amino acid sequence (AA codes and 3D coordinates of the corresponding group of atoms (N, C_α_, C, and O)) were extracted from PDB files.

Ultimately, an array of 3D coordinates was generated to describe the 3D structure of the protein. The final array of coordinates was applied to generate a graph, with each vertex representing a C_α_-atom in the main protein chain, connected by edges to the 32 nearest C_α_-atoms (KNN-graph, k = 32). Each edge and vertex of the graph contained scalar and vector features describing the 3D geometry of the protein structure. The method for determining the optimal value of k is described in the Supplementary Materials (determining the optimal value of k (nearest C_α_ atoms)).

### 3.2. Feature Extraction and Input Encoding

#### 3.2.1. Node-Level Features

**Figure 2:**
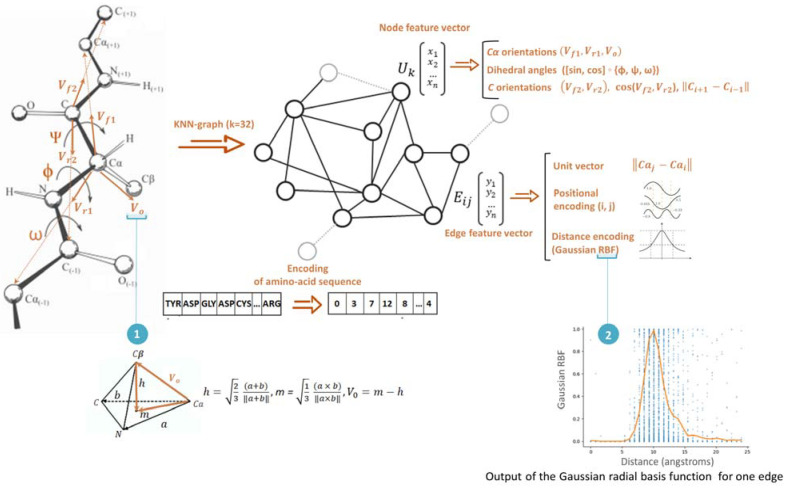
Feature extractions from protein sequences. The graph shows the protein structure.

The signs of a graph node are described by the following elements (Figure 2):

- {sin, cos} ◦ {φ, ψ, ω}, where φ, ψ, and ω are the torsion angles calculated for $C_{i-1}$*,* $N_{i}$*,* $C\alpha_{i}$, and $C_{i}$ and $N_{i+1};$
- Unit vectors of the directions to the Cα-atoms in the main chain ($\vec{V_{f1}}$*= (*$C\alpha_{i+1}$ − $C\alpha_{i}$*)* and $\vec{V_{r1}}$ = ($C\alpha_{i-1}$ − $C\alpha_{i}$));
- Unit direction vectors to the C-atom in the main chain ($\vec{V_{f2}}$ = ($C_{i+1}-C_{i}$) and $\vec{V_{r2}}$ = ($C_{i-1}$ − $C_{i}$));
- Cosines of the angles between vectors $V_{f2},\;V_{r2}$;
- The distance between the C-atom in the chain $\left\|C_{i+1}-C_{i-1}\right\|$;
- A unit vector that determines the conditional direction of the side chain (direction of the Cβ atom), $\vec{Vo}$*=*$C\beta_{i}$*–*$C\alpha_{i}$. This vector is calculated from the tetrahedral representation of the geometry of the N, Cα, and C atoms as follows:
  $$\vec{Vo}=\sqrt{\frac{1}{3}}\frac{\left(a\times b\right)}{\left\|a\times b\right\|}-\sqrt{\frac{2}{3}}\frac{\left(a+b\right)}{\left\|a+b\right\|}$$
  where vectors *a* and *b* are defined as $a=N_{i}-C\alpha_{i}b=C_{i}-C\alpha_{i}$. This vector, together with the forward and reverse vectors ($\vec{V_{f1}}$, $\vec{V_{r1}})$ determines the orientation of the amino acid residue in 3D Euclidean space.
- The amino acid sequence is encoded as a sequence of numbers (0–21).

#### 3.2.2. Edge-Level Features

Graph edge features are described by the following elements:

- a unit vector defining the direction between neighboring vertices,$\;C\alpha_{j}-C\alpha_{i}$;
- the distance between the vertices of the graph is encoded using Gaussian radial basis functions:

$$\varphi \left(r\right)=e^{-{\left(\varepsilon r\right)}^{2}}\;\left(r=\|x-x_{i}\|\right)$$

For each edge, the distance was encoded with 32 Gaussian functions, with centers uniformly spaced in the range of 0–24 Å. The edge position code (*i*, *j*) was obtained using a sinusoidal encoder, which is widely used in transformer models. This approach to the positional encoding of sequences has been previously described in detail [39].

### 3.3. Network Architecture

**Figure 3:**
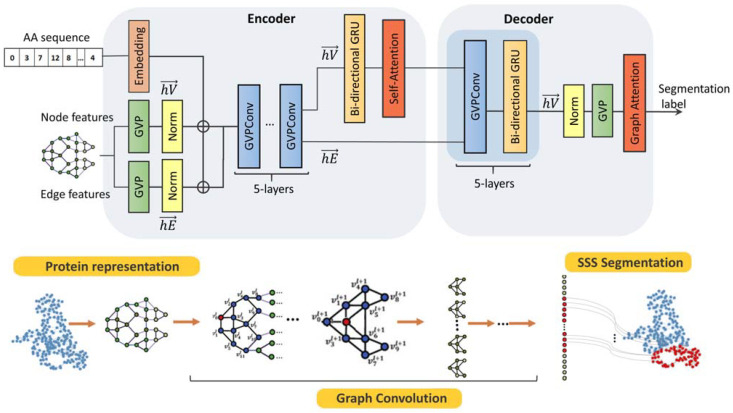
The architecture of the proposed PSSNet.

The architecture of our model is based on a combination of the geometric vector perceptron (GVP), graph neural network (GNN), and multi-layer gated recurrent unit (GRU) methods (Figure 3). The network architecture is based on the encoder–decoder principle, which is widely used in classification and segmentation problems. The encoder generates a feature map based on the input data (node position in the graph, local topology, vector, and scalar attributes of the node itself and its neighbors). The decoder extracts information from the feature map and generates classification labels for the graph nodes. The model was implemented using a binary classifier, and a separate model was trained for each SSS in the training set. As data are being accumulated, a multiclass model that works with a variety of structures has to be used in the future.

The GVP elements of the model architecture extract invariant and equivariant features from a combination of scalar and vector representations of geometric features. In addition, the GVP can approximate any continuous rotation and reflection invariant scalar function. The architecture (GVP–GNN) uses GVP modules for feature extraction and the graph convolutional network (GCN) mechanism for message, which passes between graph nodes (messaging), feature aggregation of neighboring nodes and edges, and updates node attachments during a propagation operation [40]. The GVP architecture has been previously described in detail [41].

A GVP-based neural network was used to predict amino acid sequences based on the geometric characteristics of a protein and PPI (protein–protein interaction). Because proteins are connected in sequential structures, we supplemented the model with bidirectionally controlled recurrent units (Bi-GRUs) to highlight relationships between geometric characteristics [41]. Adding GRU layers to the model significantly increased the predictive accuracy and reduced the amount of time required to train the model. Table 3 shows the architecture of the model and a brief description of the functions of the blocks.

The Adam optimizer was applied with a reduced learning rate when the accuracy metric stopped the improvement process (start with a 1 × 10^−3^ and reduce factor of 0.5). The Dice BCE loss was selected as the loss function, as it combines the Dice loss with the standard binary cross-entropy (BCE) loss, which is generally the default for segmentation models. Combining the two methods allowed for moderate diversity in the loss while improving the stability of the BCE.

### 3.4. Training and Performance Evaluation

The model training process lasted 24 epochs for each SSS and models were assessed on the validation datasets. During the learning process, the learning rate was changed from 1 × 10^−3^ to less than 1 × 10^−4^ in order to reduce the learning rate on the plateau. We used the intersection over union (IoU; also known as the Jaccard index) as the main metric for assessing the quality of model predictions. Values from 0–1 show the extent to which positions of two objects (reference [ground-truth]) predicted by the model coincide according to the following equation:

$$\mathrm{IoU}=\frac{\mathrm{Area}\;\mathrm{of}\;\mathrm{Overlap}}{\mathrm{Area}\;\mathrm{of}\;\mathrm{Union}}$$

We considered the position of SSS in the reference and predicted structures and evaluated the coincidence of their positions. The harmonic mean of the recall and precision metrics (F1) were also evaluated (Table 4).

## 4. Conclusions

Super-secondary structures are blocks of protein molecules with unique and compact spatial arrangements. Such structures are stable outside the protein globule due to pronounced hydrophobic cores. Structural biology considers SSSs as the nuclei of protein folding and as starting structures when looking for the possible folding pattern of polypeptide chains while modeling protein structures. Our model combines GNN, CNN, and RNN methods and suggests the following advantages:

- small datasets for rapid, efficient learning, and retaining;
- ability to generalize features on a relatively small training set;
- good recognition accuracy (mean IoU > 0.83);
- huge amounts of information (such as that in the PDB and AlphaFold) can be assessed within a reasonable timeframe.

Our model can classify more than 2.3 million SSSs for all protein structures available in the PDB and AlphaFold databases. The reliability and accuracy of the model were demonstrated on four types of SSSs taken from the public Structural Elements Database (PSSKB, https://psskb.org/, accessed on 28 October 2022); however, the model is generic and can be applied to a wider set of SSS types. The assembled set of SSS structures opens up new options for studying the uniqueness and compactness of protein spatial packing and folding nuclei, and can also act as starting structures for searching for possible polypeptide chain folding while modeling protein structures.

Future efforts will target the diversity of SSS types (Greek key, Rossmann fold, etc.) in the segmentation model and replenishing the database. We will also focus on improving annotations and ensuring the quality of SSS presentations. Furthermore, we will generate sufficient information for users with extensive experience in structural biology and new entrants into that. We will also tailor the database to meet the needs of the research community and provide accurate SSS information for future updates.

## Figures and Tables

**Table 1:** Number of SSSs recognized in open knowledge databases (PDB and AlphaFold).

SSS	PDB (185,469 Structures)	AlphaFold (2021) (360,000 Structures)
βαβ-unit	461,336	233,882
α-hairpin	390,965	563,946
β-hairpin	360,845	280,181
αα-corner	5977	8153

**Table 2:** Comparison between our proposed and other models (mean IOU).

SSS	PSSNet		CurveNet		DGCNN	
	Train	Val	Train	Val	Train	Val
βαβ-unit	0.928	0.894	0.742	0.697	0.691	0.656
α-hairpin	0.964	0.957	0.814	0.795	0.731	0.688
β-hairpin	0.998	0.983	0.845	0.833	0.749	0.711
αα-corner	0.933	0.991	0.781	0.732	0.621	0.571

**Table 3:** Model architecture and implementation details.

Block	Layer	Description
Encoder	Embedding	Words in AA-sequence using a dense vector form.
	GVP	Module for learning vector- and scalar-valued functions over geometric vectors and scalars.
	Norm	Layer normalization for vector features (L2-normalization).
	GVPConv (5-layers)	Implements GVP transforms and uses message-passing mechanisms from neighboring nodes and edges to aggregate a function of hidden states and update node embedding at each graph propagation step.
	Bi-GRU (2-layer module)	Recurrent unit with input and forget gates. The Bi-GRU considers two separate sequences: from right to left and vice-versa. We considered the sequence of the hidden states of the node features of the graph.
	Self-attention	This mechanism allows the discovery of connections between elements of the input sequence and the selection of those required for future generations [42]. We considered the sequence of the hidden states of the node features of the graph.
Decoder	GVPConv + Bi-GRU (5-layers)	Decoder block to reconstruct and obtain the graph structure from the encoder’s hidden state.
	GVP	Last GVP module.
	Multi-head graph attention	This module has a one-way scalar sigmoid output to predict node labels [43].

**Table 4:** Performance metrics: IoU and F1.

SSS	Mean IoU (Training)	Mean IoU (Validation)	F1
βαβ-unit	0.928	0.894	0.978
α-hairpin	0.964	0.957	0.984
β-hairpin	0.998	0.983	0.991
αα-corner	0.933	0.991	0.994

## Data Availability

The code source designed for super-secondary structures classification (PSSNet) has been deposed to the open-access GitHub resource and is available at the following link: https://github.com/Denis21800/PSSNet.

## Supplementary Materials

The supporting information can be downloaded at: https://www.mdpi.com/article/10.3390/ijms232314813/s1. Reference [44] is cited in the supplementary materials.
