# Supplementary material for: PSSNet—An Accurate Super-Secondary Structure for Protein Segmentation
Source: Int J Mol Sci. 2022 Nov 26;23(23):14813. doi: 10.3390/ijms232314813 (PMC9740782; doi:10.3390/ijms232314813)
Supplement: Supplementary file 1 [file ijms-23-14813-s001.zip › ijms-2029795-supplementary.pdf]

Datasets definition

Datasets used in the study and accompanied annotations are published and submitted at: https://doi.org/10.6084/m9.figshare.21529812.v1

The βαβ-motif

The dataset was enrolled from protein structures, most of which are represented by Alpha-Beta classes according to the CATH classification (3.40 3-layer αβα Sandwich and 3.40 Alpha-Beta Barrel). Structural sequences of interest Coil→β-Strand→Coil were extracted from α-helix→Coil→β-Strand→Coil (Figure S1).

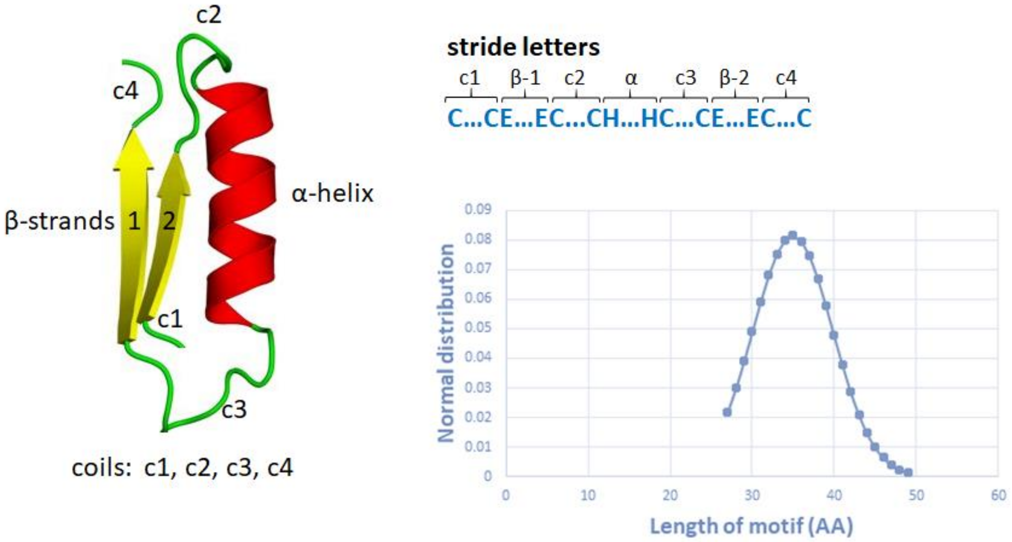

Figure S1. Structure of the βαβ motif and distribution of initial data according to the CATH classifier.

Motifs with different lengths of constituent elements were selected to design the dataset of essential βαβ motifs (Table S1 and Figure S2) and the included structures were manually curated for the compliance with βαβ motifs specificities.

Table S1. The distribution of the lengths of the βαβ motif elements.

Structure element	Length (AA)				
	min	max	median	mean	SD
Total	27	49	34	34.9	4.9
α-helix	4	29	12	12.2	3.2
β-strand (1)	2	15	4	4.3	1.7
β-strand (2)	2	10	4	4.3	1.4
c1	1	9	2	2.48	1.3
c2	0	25	4	4.4	2.88
c3	0	20	3	2.9	1.9
c4	0	8	2	2.3	0.9

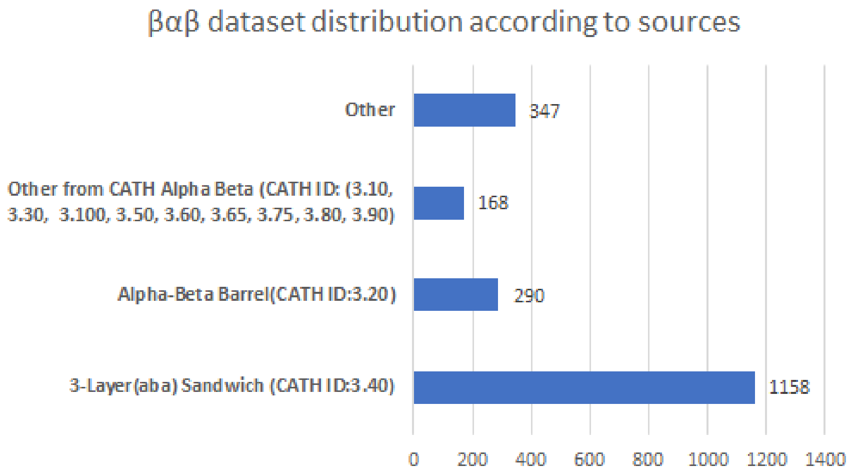

Figure S2. βαβ dataset distribution according to sources.

A set of negative samples was approached by the following:

1. 51% of the collected structures were randomly selected from distinct types of structures (α-hairpin, β-hairpin, αα-corner; 700 structures of each type).
2. 49% of structures were randomly selected from the PDB structures with length of 25-50 AA residues but not containing βαβ motifs (the process was controlled by STRIDE line and by experts for the absence of βαβ).

The final dataset was divided into training, test and validation subsets by random sampling (Table S2).

Table S2. Distribution of positive and negative examples of training, test and validation sets for βαβ motif.

Positive examples		Negative examples	
Total	1963	Total	4000
Training set	1063	Training set	2000
Test set	500	Test set	1000
Validation set	400	Val set	1000

The β-hairpin motif

The dataset of β-hairpin motif was shaped from protein structures, a significant part of which is represented by the mainly Beta and Alpha-Beta classes according to the CATH classification (CATHID: 2.xx, 3.xx). Structural sequences of Coil→β-Strand type were extracted from Coil→β-Strand→Coil sequences (Figure S3).

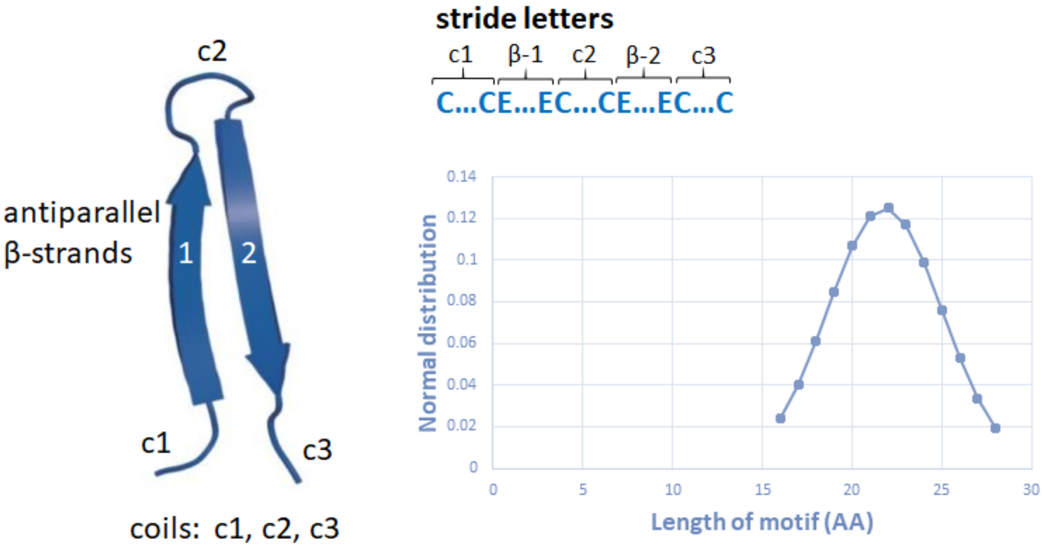

Figure S3. Structure of the β-hairpin motif and distribution of initial data according to the CATH classifier

Table S3. The distribution of the lengths of the β-hairpin motif elements.

Structure element	Length (AA)				
	min	max	median	mean	std
Total	16	28	22	21.8	3.19
β-strand (1)	2	11	6	5.9	1.85
β-strand (2)	2	11	6	5.9	1.87
c1	1	7	2	2.1	0.7
c2	1	16	4	3.8	2.72
c3	1	7	3	2.9	0.92

The training dataset was designed from motifs containing different lengths of constituent elements selected from the total sample (Table S3 and Figure S4), and the final set of structures was curated by experts for the compliance with the β-hairpin motif.

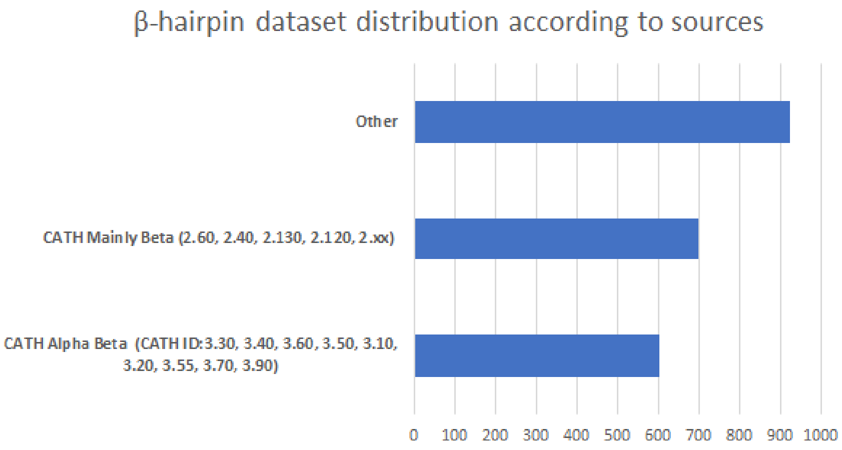

Figure S4. β-hairpin dataset distribution according to sources.

Negative samples were approached as following:

1. 50% of structures were randomly selected from structures of distinct types (βαβ, α-hairpin, αα-corner; 700 structures of each type).

2. 50% of the structures were randomly selected from the PDB among sequences with length ranged within 16-35 AA residues and not containing β-hairpin motifs (the verification was carried out using the STRIDE line and controlled by experts for the absence of β-hairpin motifs).

The designed dataset was split into training, test, and validation subsets by random sampling (Table S4).

Table S4. Distribution of positive and negative examples of training, test and validation sets for β-hairpin motif.

Positive examples		Negative examples	
Total	2225	Total	4000
Training set	1225	Training set	2000
Test set	500	Test set	1000
Validation set	500	Val set	1000

The α-hairpin motif

The dataset of the α-hairpin motif is formed from protein structures, a significant part of which is represented by the Mainly Alpha and Alpha-Beta classes according to the CATH classification (CATHID:1.xx, 3.xx), structural sequences of the Coil→α-helix→Coil type were extracted →α-helix→Coil. The distribution of initial data according to the CATH classifier is presented in Figure S5. Table S5 shows the distribution of the lengths of the elements that make up the α-hairpin motifs in the dataset.

To form a training data set, motifs containing different lengths of its constituent elements were selected from the total sample.

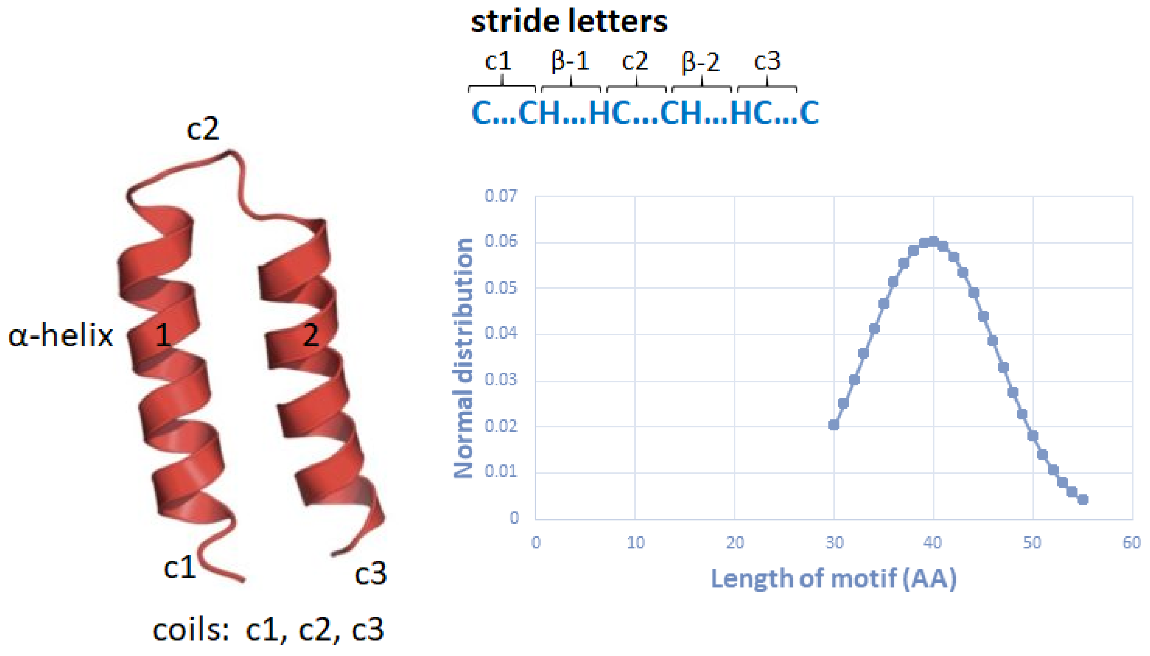

Figure S5. Structure of the α-hairpin motif.

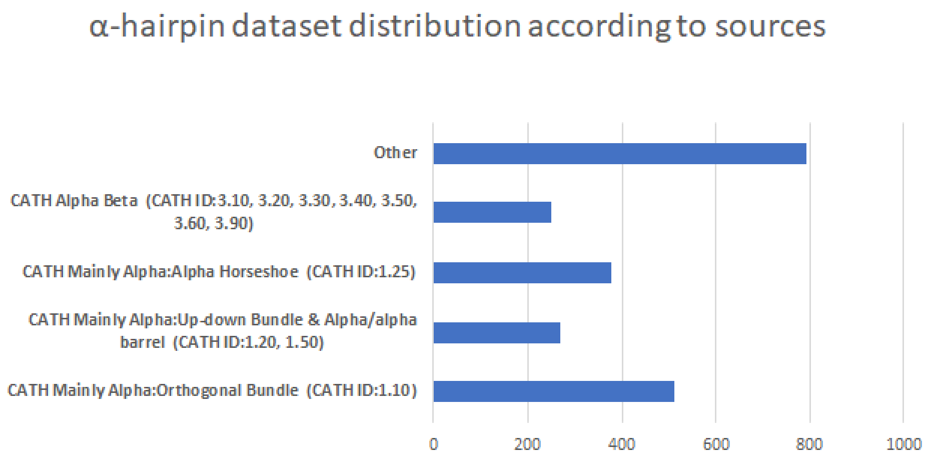

Figure S6. α-hairpin dataset distribution according to sources.

Table S5. The distribution of the lengths of the α-hairpin motif elements.

Structure element	Length (AA)				
	min	max	median	mean	std
Total	30	55	39	39.74	6.6
α-helix (1)	4	35	14	14.48	4.97
α-helix (2)	4	37	15	15.24	4.99
c1	0	5	2	1.93	0.3
c2	1	27	5	5.18	3.04
c3	1	3	3	2.89	0.4

Structures included in the dataset were verified by experts for the compliance to criteria of α-hairpin motif. Negative training sample was approached as following:

1. 50% of structures were randomly selected from structures of distinct types (βαβ, β-hairpin, αα-corner; 700 structures of each type).
2. 50% of structures were randomly selected from PDB bank if length is ranged between 25-60 AA residues and sequences do not contain α-hairpin motifs (control was managed by using the STRIDE line and by experts for the absence of α-hairpin motifs).

   The collected dataset was split into training, test and validation subsets by random sampling (Table S6).

Table S6. Distribution of positive and negative examples of training, test and validation sets for α-hairpin motif.

Positive examples		Negative examples	
Total	2200	Total	4000
Training set	1200	Training set	2000
Test set	500	Test set	1000
Validation set	500	Val set	1000

The αα-corner motif

The dataset of the αα-corner motif was collected from protein structures, a significant part of which is represented by the Mainly Alpha as the Orthogonal Bundle class according to the CATH classification (CATHID:1.10), structural sequences of which are ordered as Coil→α-

helix→Coil→α-helix→Coil, so that the angle between the projection planes of helices is between 70-90°. The main task of the collected set of structures is to test the ability of the model to recognize the utter topology of structures, considering spatial relationships between the objects of the structure (Figure S7).

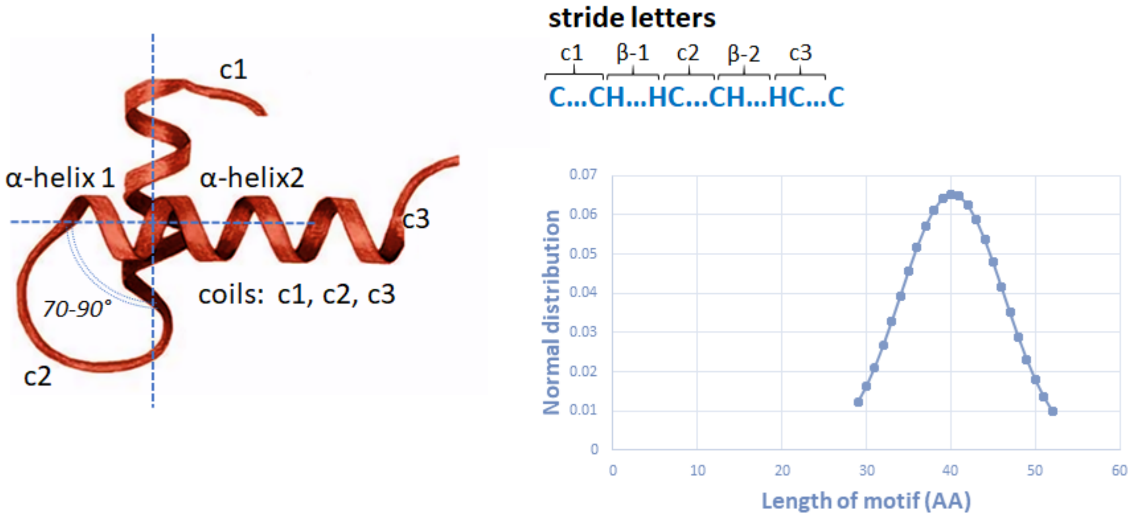

Figure S7. Structure of the αα-corner motif and distribution of initial data according to the CATH classifier.

The training dataset was collected from motifs with different lengths of constituent elements, which were selected from the total sample (Table S7 and Figure S8).

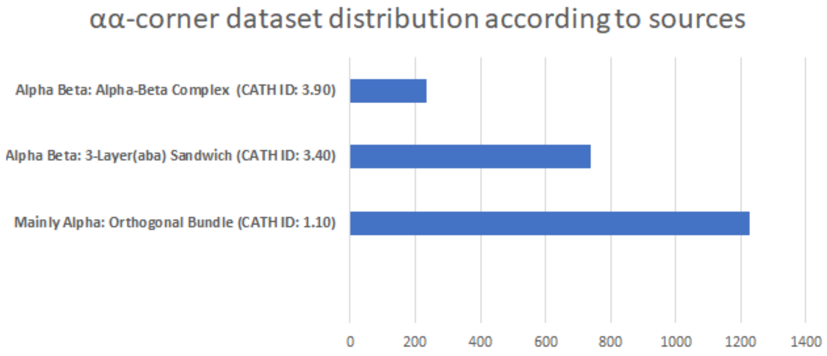

Figure S8. αα-corner hairpin dataset distribution according to sources.

Table S7. The distribution of the lengths of the αα-corner motif elements.

Structure element	Length (AA)				
	min	max	median	mean	std
Total	29	52	37	40.18	6.11
α-helix (1)	6	35	16	17.15	4.96
α-helix (2)	6	32	12	14.15	4.42
c1	0	10	0	0.84	1.2
c2	1	15	6	6.35	2.39
c3	1	10	1	1.67	1.06

Structures included in the dataset were verified by experts for the compliance with the αα-corner motif criteria. The negative training sample was approach as following:

1. 50% of structures were randomly selected from structures of distinct types (βαβ, β-hairpin, α-hairpin; 700 elements of structures of each type).
2. 50% of the structures were randomly selected from the PDB if sequences fall in length within 25-50 AA residues and do not contain αα-corner motifs.

The designed dataset was split into training, test and validation subsets (Table S8).

Table S8. Distribution of positive and negative examples of training, test and validation sets for αα-corner motif.

Positive examples		Negative examples	
Total	2195	Total	4000
Training set	1188	Training set	2000
Test set	500	Test set	1000
Val set	500	Val set	1000

Model evaluation metrics

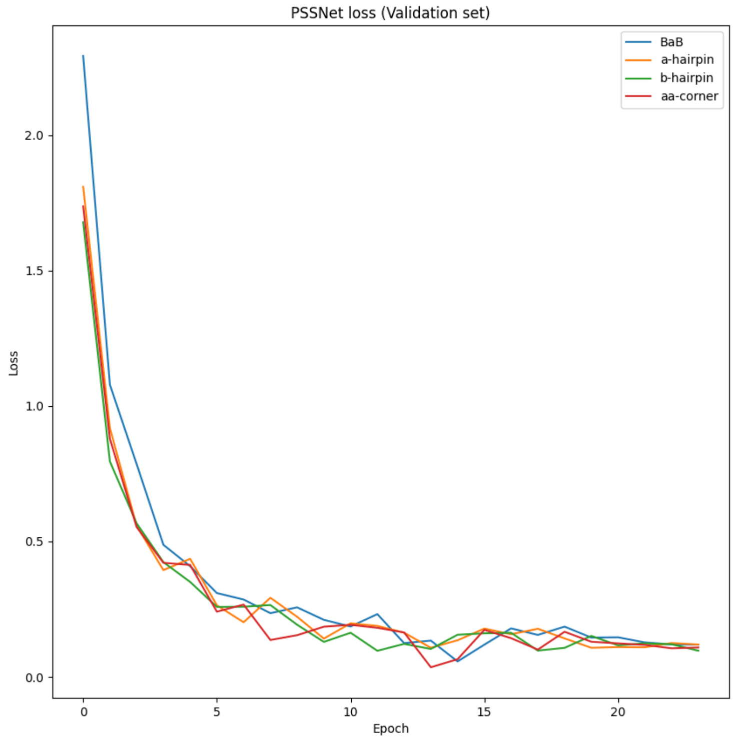

Figure S9. PSSNet: Evaluated losses by epoch on validation set (βαβ-unit, α-hairpin β-hairpin, αα-corner).

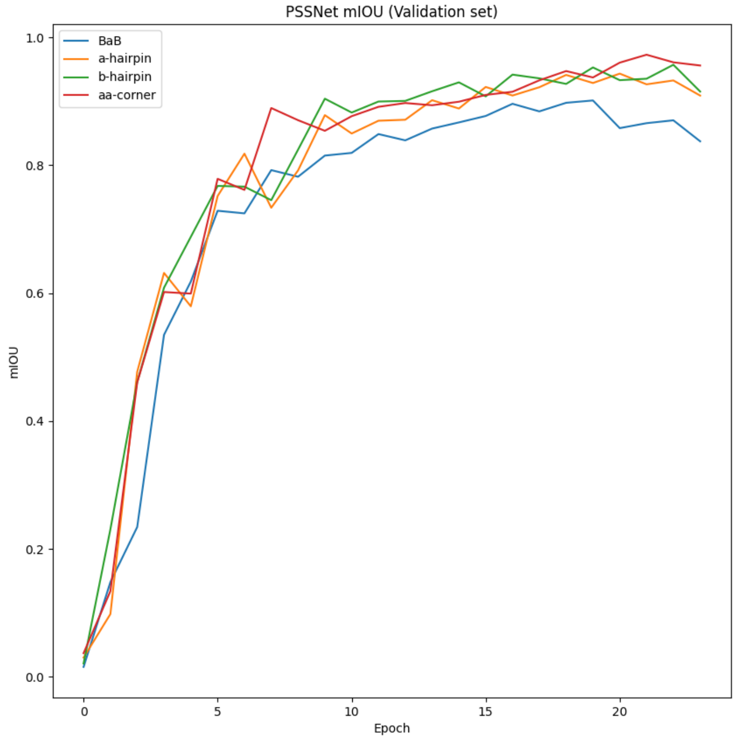

Figure S10. PSSNet: Evaluated mean of IOU by epoch on validation set (βαβ-unit, α-hairpin β-hairpin, αα-corner)

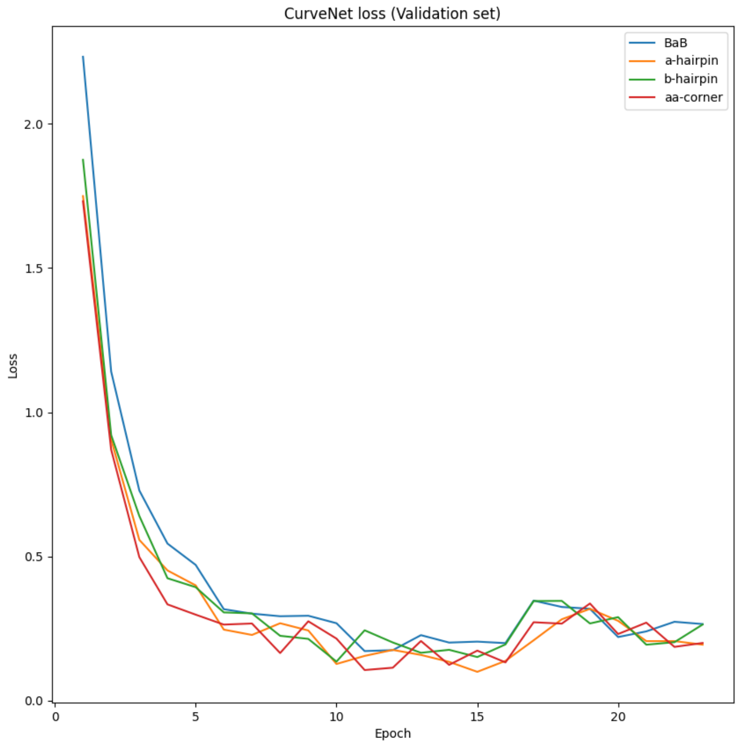

Figure S11. CurveNet: Evaluated losses by epoch on validation set (βαβ-unit, α-hairpin β-hairpin, αα-corner).

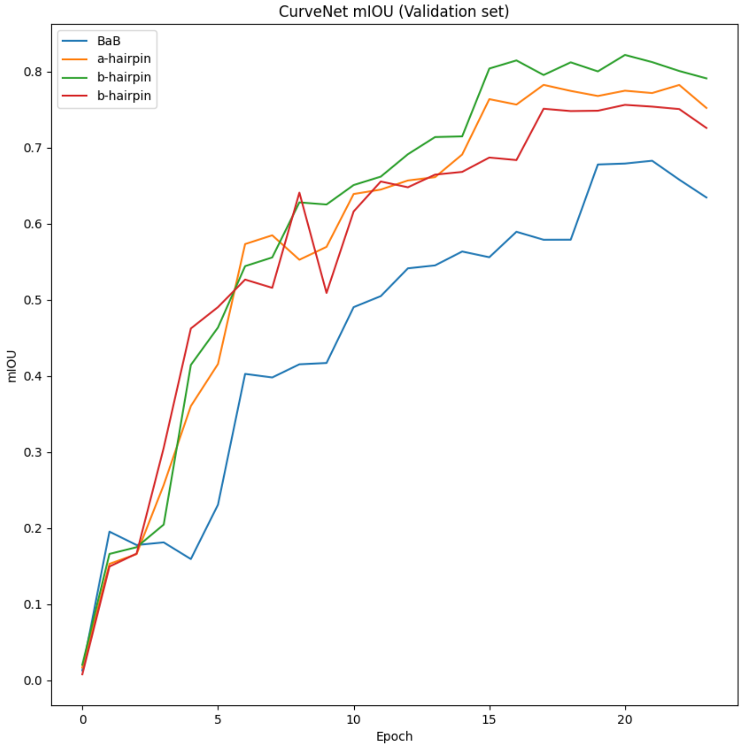

Figure S12. CurveNet: Evaluated mean of IOU by epoch on validation set (βαβ-unit, α-hairpin β-hairpin, αα-corner)

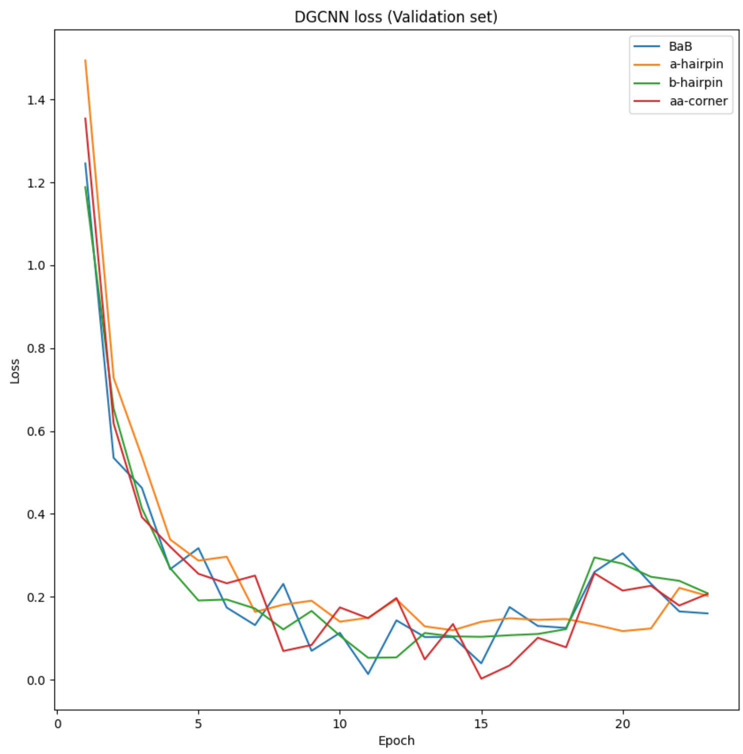

Figure S13. DGCNN Evaluated losses by epoch on validation set (βαβ-unit, α-hairpin β-hairpin, αα-corner).

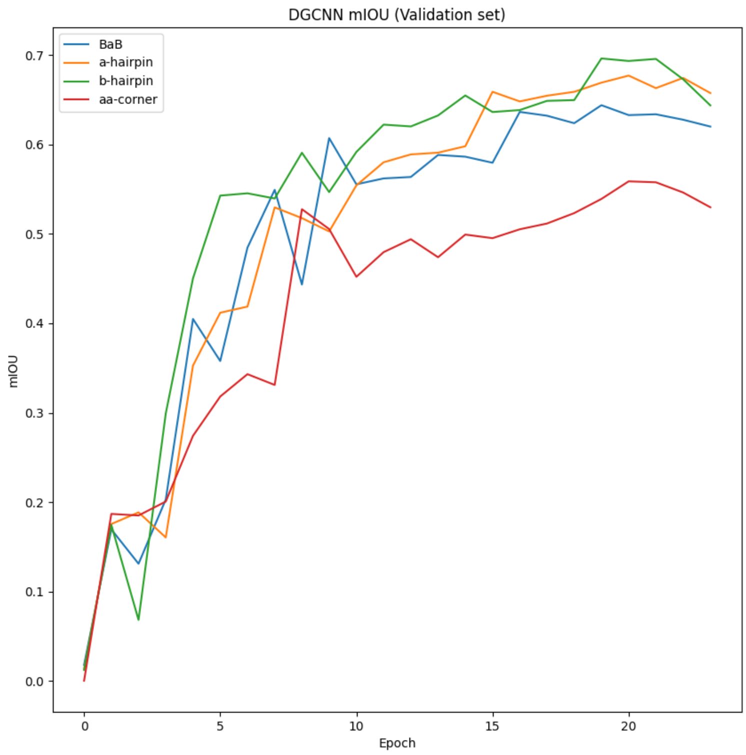

Figure S14. CurveNet: Evaluated mean of IOU by epoch on validation set (βαβ-unit, α-hairpin β-hairpin, αα-corner)

GPU memory usage

Memory usage graphs. Obtained from the results of profiling the model (Pytorch 1.10.1, Pytorch profiller).

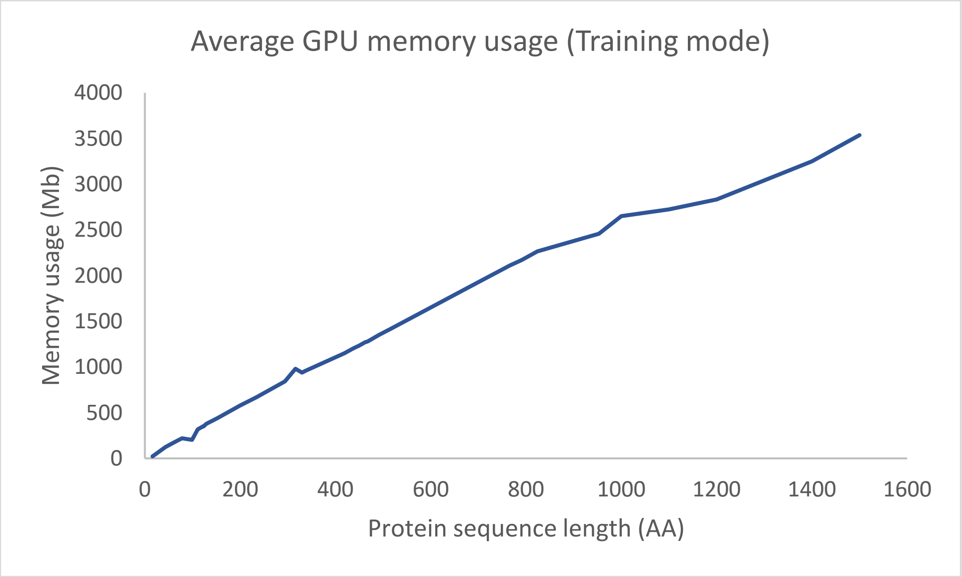

Figure S15. PSSNet: GPU memory usage versus protein sequence length (Training mode)

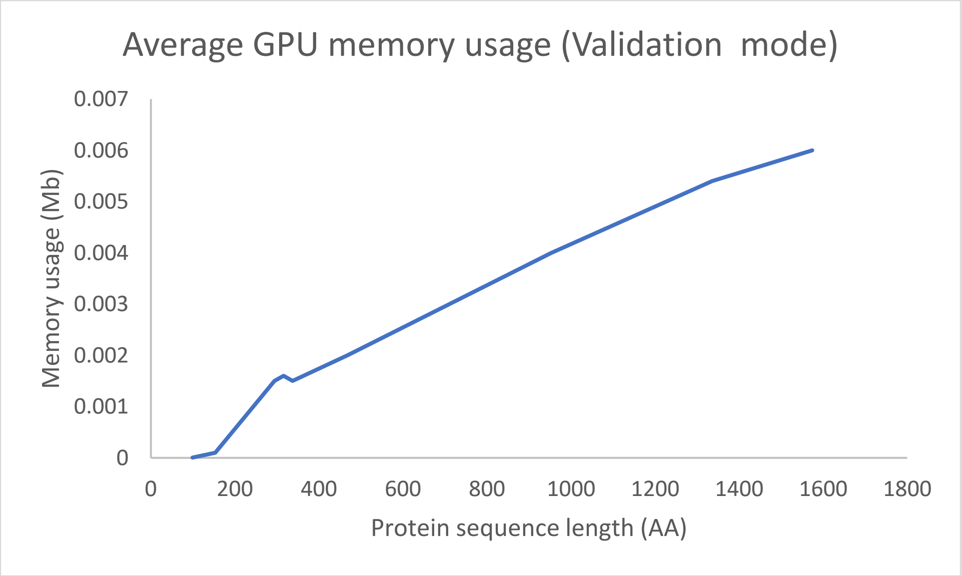

Figure S16. PSSNet: GPU memory usage versus protein sequence length (Validation mode)

Determining the optimal value of k (nearest C-alpha atoms)

Value *k*=32 defines level of nearest neighbors' geometrical information aggregation on graph nodes. In general, nearest neighbors are not always contain all the current SSS atoms. For every layer of a model graph node aggregates information about its neighboring nodes. Example of such aggregation is shown on Fig. 17.

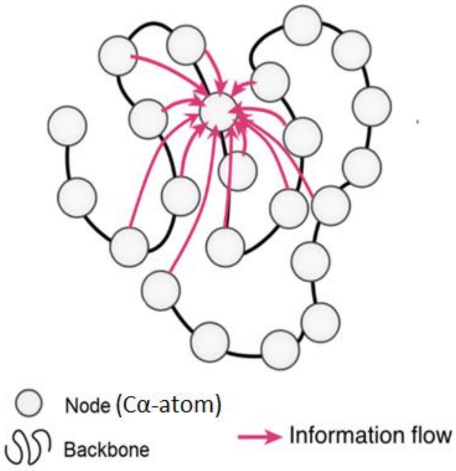

Figure S17. Information flow for structures encoder of PSSNet model.

Figure S18 Shows an example of graph for single protein (PDB:2ATZ). As this example shows, this is strongly connected graph, and the network consists of five aggregating layers. This way every node has information not only about its neighbors but about topology of the whole structure.

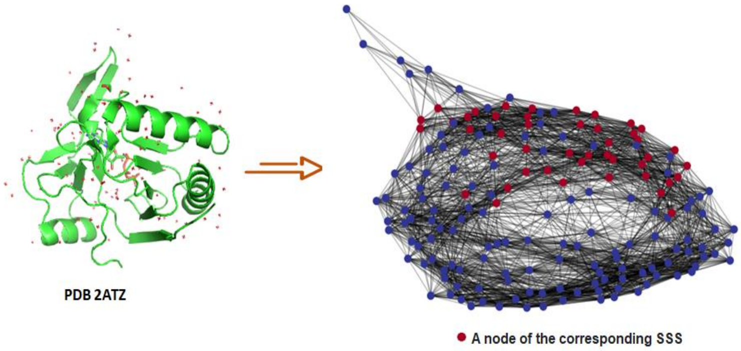

Figure S18. An example of a graph built for a protein from the training set (PDB ID structure - 2ATZ).

To define optimal value for *k* parameter («nearest neighbors' amount») we studied training parameters of known Protein MPNN network which is similar to PSSNet by its principles while solving Inverse Folding problem [1].

Authors conducted a study and proved that optimal value is in rage from 32 to 48 [1].

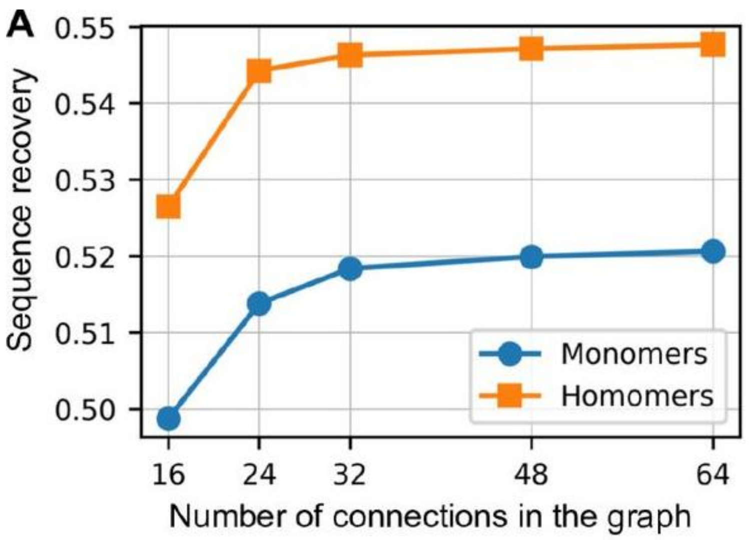

Figure S19. ProteinMPNN network: Sequence recovery as a function of the number of nearest neighbors in the graph [1]

We conducted similar research with *k* values 24, 32, 36 and 48. We found out that if *k*=24, precision of model is significantly lower while training becomes faster. Increasing the value gives no significant precision increase. Also increasing of value raises RAM consumption. Plot of mean IOU depending on *k* on βαβ dataset is shown on Figure S20 (other datasets shows the same picture). Plot of consumed GPU RAM depending on k is on Figure S21.

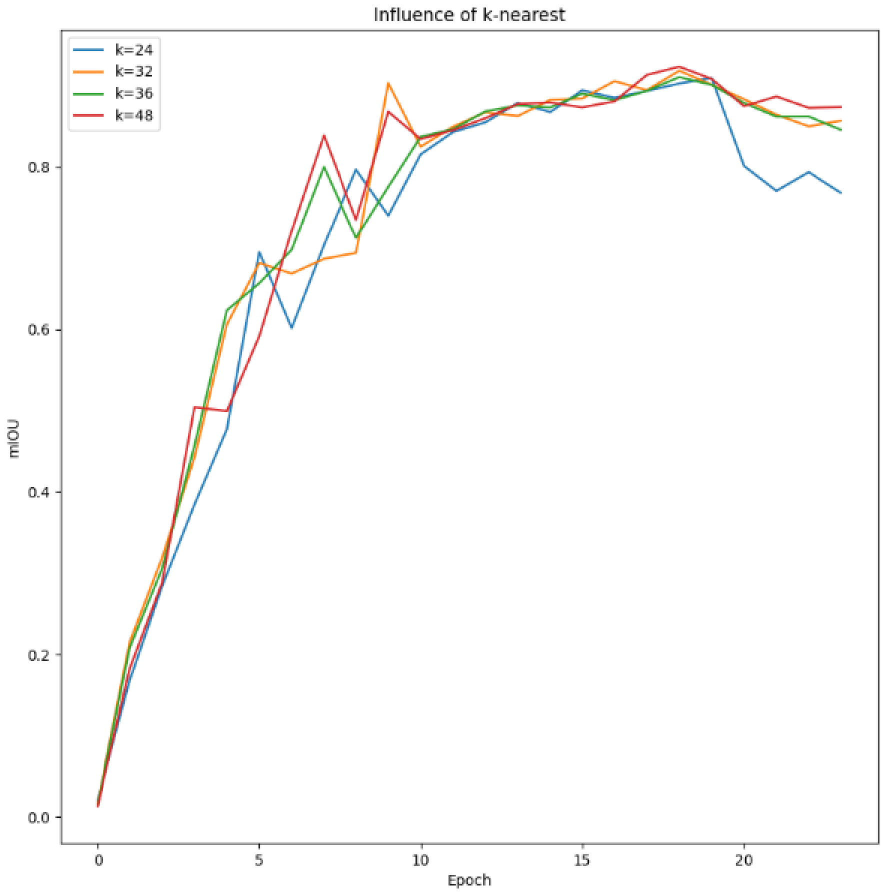

Figure S20. PSSNet: Mean intersection over union (mIOU) on the number of connections in the graph (k).

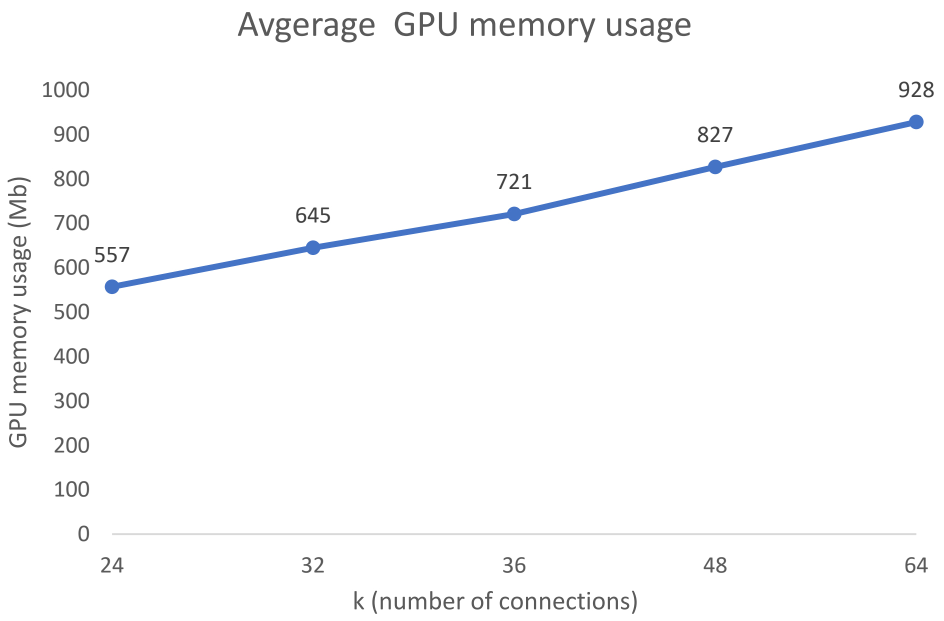

Figure S21. PSSNet: GPU Memory usage on the number of connections in the graph (k).

This is the reason we decided k=32 as we needed to process large amount of data containing long sequence. Small increase of precision with higher k value has significant cost in memory consumption what was not acceptable for us.

References

1. Dauparas, J., Anishchenko, I., Bennett, N., Bai, H., Ragotte, R. J., Milles, L. F., Wicky, B. I. M., Courbet, A., de Haas, R. J., Bethel, N., Leung, P. J. Y., Huddy, T. F., Pellock, S., Tischer, D., Chan, F., Koepnick, B., Nguyen, H., Kang, A., Sankaran, B., Bera, A. K., … Baker, D. (2022). Robust deep learning-based protein sequence design using ProteinMPNN. Science (New York, N.Y.), 378(6615), 49–56. https://doi.org/10.1126/science.add2187.
